# Machine Learning Based Microbiome Signature to Predict Inflammatory Bowel Disease Subtypes

**DOI:** 10.3389/fmicb.2022.872671

**Published:** 2022-05-17

**Authors:** Jose Liñares-Blanco, Carlos Fernandez-Lozano, Jose A. Seoane, Guillermo López-Campos

**Affiliations:** ^1^Department of Computer Science and Information Technologies, Faculty of Computer Science, CITIC, University of A Coruña, A Coruña, Spain; ^2^GENYO, Centre for Genomics and Oncological Research, Pfizer/University of Granada/Andalusian Regional Government PTS Granada, Granada, Spain; ^3^Department of Statistics and Operational Research, University of Granada, Granada, Spain; ^4^Vall d'Hebron Institute of Oncology, Barcelona, Spain; ^5^Wellcome-Wolfson Institute for Experimental Medicine, Queen's University Belfast, Belfast, United Kingdom

**Keywords:** machine learning, feature selection, inflammatory bowel disease, microbiome, Crohn's disease, ulcerative colitis

## Abstract

Inflammatory bowel disease (IBD) is a chronic disease with unknown pathophysiological mechanisms. There is evidence of the role of microorganims in this disease development. Thanks to the open access to multiple omics data, it is possible to develop predictive models that are able to prognosticate the course and development of the disease. The interpretability of these models, and the study of the variables used, allows the identification of biological aspects of great importance in the development of the disease. In this work we generated a metagenomic signature with predictive capacity to identify IBD from fecal samples. Different Machine Learning models were trained, obtaining high performance measures. The predictive capacity of the identified signature was validated in two external cohorts. More precisely a cohort containing samples from patients suffering Ulcerative Colitis and another from patients suffering Crohn's Disease, the two major subtypes of IBD. The results obtained in this validation (AUC 0.74 and AUC = 0.76, respectively) show that our signature presents a generalization capacity in both subtypes. The study of the variables within the model, and a correlation study based on text mining, identified different genera that play an important and common role in the development of these two subtypes.

## 1. Introduction

The microbiota consists of about 100 trillion commensal microorganisms with main roles in metabolic processes in the host. Therefore, decoding the impact of the microbiota on human health and disease is currently one of the greatest challenges in biomedicine.

A substantial body of evidence supports a relevant role of the microbiota in inflammatory bowel disease (IBD) (Franzosa et al., 2019; Lloyd-Price et al., 2019; Amoroso et al., 2020; Ananthakrishnan, 2020; De Musis et al., 2020; Glassner et al., 2020; Haifer et al., 2020; Aldars-Garćıa et al., 2021), including ulcerative colitis (Guo et al., 2020) and Crohn's disease (Scanlan et al., 2006). Inflammatory bowel disease (IBD) is a chronic complex disease of the gastrointestinal tract. Patients with IBD can experience a wide range of symptoms, but the pathophysiological mechanisms that cause these individual differences in clinical presentation remain largely unknown. Therefore, great emphasis has been placed on the effect of specific taxa and their metabolites to explain the microbial influence on IBD development as well as to identify clinical targets for innovative treatments (Nishida et al., 2018).

Great efforts have been made at the international level to provide the scientific community with metagenomic datasets in very large populations. The main example was the emergence of the Human Microbiome Project (Huttenhower et al., 2012; Methé et al., 2012), with the aim of characterizing the human microbiome and analyzing its role in human health and disease. Moreover, in 2012 the AGP (McDonald et al., 2018) was launched as a collaboration between the Earth Microbiome Project (EMP) and the Human Food Project (HFP) focused on characterizing global microbial taxonomic and functional diversity as well as understanding microbial diversity across human populations.

Nowadays, thanks to these and other initiatives, numerous works have reported different techniques to identify relevant metagenomic genera and/or species for stratification and classification of patients according to their disease (Bai et al., 2019; Boolchandani et al., 2019; Aryal et al., 2020; Bezek et al., 2020; Fernández-Edreira et al., 2021).

However, more complex diseases without a clear etiopathology have yet to be further explored. In the case of IBD, there are few papers that have hosted Machine Learning-based (ML) analysis for the identification of new genera that may play a key role in the development of the disease. In addition, due to the characteristics of metagenomic data, high sparsity and high dimensionality, a robust methodology must be used for processing and training algorithms.

In this paper we focused in the application of different ML algorithms and we present the results obtained after analyzing and training them with metagenomic data downloaded from the AGP. The models were trained for the classification of samples according to their IBD diagnosis, without specifying the type of disease. Different feature selection procedures were used to identify those genera presenting significant differences. Finally, the best model was taken to external validation in two publicly available cohorts, for the identification of Crohn's disease and ulcerative colitis. The best model achieved at this stage performances higher than AUC = 0.7 in both datasets, showing a high generalization of the model.

## 2. Materials and Methods

### 2.1. Training Dataset

The data used in this work was downloaded from the American Gut Project (AGP). We have created a public repository where we indicate all the steps to proceed for the data download: https://github.com/jlinaresb/IBDpred. Raw data of Operational Taxonomic Unit (OTUs) counts from AGP were downloaded. Annotation of taxonomic data and other aspects related with data generation can be consulted in original paper (McDonald et al., 2018).

### 2.2. Preprocessing Pipeline

Since some patients had multiple samples, a single sample from each patient was selected first. The selection was made according to the sampling date, selecting the most recent one. For those patients in whom it was not possible to make the selection in this way, the selection was made randomly.

Phyloseq R package (McMurdie and Holmes, 2013) was used to manage this data. Phyloseq class was created from biom, tree and clinical files. Only fecal samples were selected to further analysis. We obtained a total of 36.405 OTUs from 12.189 individuals. The first step was agglomerate all OTUs at the taxonomic rank of Genus. After this step, the dataset was simplified to 2.082 OTUs. Those OTUs that had an unknown genus (labeled as “g__”) were eliminated. Finally, the dataset was reduced to 1,322 variables. Then, it was carried out an analysis of outliers using the Isolation Forest technique (Liu et al., 2008) from H20 R package (LeDell et al., 2020). With this technique we were able to eliminate a total of 1.219 individuals. The remaining individuals were labeled according IBD diagnosis. The dataset was labeled and balanced to the positive class. For our analyses we focused in patients with IBD diagnosis, regardless of the subtype of the disease. The final dataset presented 642 individuals. Control samples were selected randomly between those without the disease. Then, OTU's counts were log2 normalized before feature selection and machine learning analysis.

After preprocessing, whole dataset was splitted into 85% train and 15% test set. Train set was the input to feature selection algorithm.

Characteristics of both train and test data are showed in Table 1. *P*-values were calculated in order to compare different subgroups of patients according the confounders.

**Table 1:** Summary descriptives table by groups of “cohort.”

	**Test**	**Train**	**p.overall**
	***N* = 97**	***N* = 545**	
Age	46.7 (17.8)	45.1 (17.0)	0.413
Sex			0.267
Female	50 (51.5%)	286 (52.5%)	
Male	45 (46.4%)	251 (46.1%)	
Unknown	1 (1.03%)	8 (1.47%)	
Unspecified	1 (1.03%)	0 (0.00%)	
IBD			0.270
Control	54 (55.7%)	267 (49.0%)	
IBD	43 (44.3%)	278 (51.0%)	

### 2.3. Feature Selection

In order to select the best features to discriminate samples according IBD diagnosis, several feature selection processes were applied to reduce the dimensionality of the problems and remove noisy features, present in several biological problems. Since there is no standard for the selection of features on metagenomic data, which are characterized by being extremely sparse, a search was performed using several feature selection techniques. Each of the techniques used is discussed below.

#### 2.3.1. Kruskal-Wallis Tests

In this case, we have used a filter approach to obtain a score that measures the relevance of the features against the class vector by observing only the intrinsic properties of the data without taking any assumptions from the classifiers. This approach is computationally simple and fast. For the calculation of the relevance of the variables, a kruskal-wallis test was used. Because characteristics of the dataset, a non-parametric univariate statistical tests was used. According to the significance of this test, we ranked the features and explored the sizes of different subsets (5, 10, 20, and 40).

#### 2.3.2. Fast Correlation Based Filter for Feature Selection (FCBF)

Also, a predominant correlation analyisis (Yu and Liu, 2003) was used to evaluate features correlation train dataset and to filter out the most informative features, reducing the dimensionality of the analysis. This approach is basically a multivariate filtering method, which uses the measure of entropy (H) and the Information Gain (IG) for the search of the subgroup of dominant features for a specific condition. The action of these two measures is encapsulated in the Symmetrical Uncertainty (SU) (Press et al., 2007).

Initially, the SU value was calculated for each feature, keeping relevant features based on a threshold (0.0025) and sorting them in descending order according to this value. Secondly, features providing redundant information were removed. For a better understanding of this methodology, see Yu and Liu (2003). Thus, we selected features in a model-independent manner, selecting features with high correlation with patient country origin, but little correlation with other non-informative features (predominant correlation). In our study, this approach was run on the entire set of features, after preprocessing, with more than 1.000 different features. Out of these, the algorithm extracted 37 that satisfied the defined requirements.

#### 2.3.3. Linear Decomposition Model (LDM)

Linear decomposition model (Hu et al., 2021) was used to investigate association of the metagenomic profile with IBD diagnosis. LDM provides both global test of any effect of the microbiome and tests of the effects of individual OTUs with false discovery rate (FDR)-based correction for multiple testing. Taxa with differential abundance across sample groups were detected by LDM with FDR correction (FDR nominal = 0.01) using the Benjamini-Hochberg method. We have paid attention to which OTUs had significant differences in abundance between IBD and non-IBD samples. After applying the model, we found out three OTU's with significant difference.

The model was carried out establishing a maximum of 10,000 permutations as stopping criteria and the Bray Curtis method was used to calculate the distance matrix.

#### 2.3.4. Differential Abundance

For this approach carried out a differential analysis using the (Robinson et al., 2010) package. This package was first implemented to model gene expression data, such as RNASeq. In this work, we have used the adaptation of edgeR for metagenomic data (McMurdie and Holmes, 2014), implemented in the phyloseq package (McMurdie and Holmes, 2013). To estimate differential expressed OTU's we used a Fisher exact test. Finally, through this approach, we select a total of 14 significant OTU's.

### 2.4. Machine Learning

Machine Learning helps to explain and extract specific knowledge from a set of data that humans would not be able to achieve. In this work, we used two different implementations of the following of Machine Learning algorithms: random forest (RF) (Breiman, 2001) and generalized linear model (glmnet) (Friedman et al., 2010).

The critical part of any Machine Learning algorithm is its training. Each algorithm has a set of hyperparameters that must be tuned to fit the training data. The methodology used for model validation will be explained in detail later.

Random forest (RF) was developed by Breiman (2001) and consists of an ensemble of independent decision trees based on random resampling of the variables for the construction of each tree. A majority vote of the trees in classification is taken as the prediction. Thus, RF adds an additional layer of randomness to a conventional bagging approach.

A search was made of the appropriate values for the parameters mtry (number of variables randomly sampled in each division of the data) and nodesize (minimal size of the terminal nodes). The range for the number of variables was established between 1 and, as the upper limit, the square root of the number of variables with the largest dataset. The minimal size of the terminal nodes ranged between 1 and 3. Low values for this parameter provide great growth and depth of each tree, improving the accuracy of predictions. In addition, the number of trees was 1,000. A large number of trees ensures that each observation is predicted at least several times.

Logistic regression is a popular classification algorithm in machine learning problems when the response variable is categorical. The logistic regression algorithm represents the class-conditional probabilities through a linear function of the predictors. In this study, we use a fast regularization algorithm that fits a generalized linear model with elastic-net penalties, called glmnet. The algorithm was developed by Friedman et al. (2010). The elastic-net penalty can tend toward the lasso penalty (Tibshirani, 1996) to the ridge penalty (Saunders et al., 1998). The ridge penalty is known to shrink the coefficients of correlated predictors toward each other, while the lasso tends to pick one of them and discard the others. Therefore, the elastic-net penalty mixes these two.

The grids of alpha and lambda for tuning are (0.0001, 0.001, 0.01, 0.1, and 1) and (0, 0.15, 0.25, 0.35, 0.5, 0.65, 0.75, 0.85, and 1), respectively. Alpha controls the elastic-net penalty, from lasso (α = 1) to ridge (α = 0). The lambda parameter controls the total force of the penalty.

### 2.5. Experimental Design

The experimental design focused on the search for metagenomic variables for the stratification of patients according to IBD diagnosis. The AGP dataset was used for this purpose. The AGP comprises the largest dataset in terms of metagenomics research. It consists of more than 16,000 samples from individuals from all over the world, although with the highest density from the USA, Canada and the United Kingdom. This dataset, due to its diversity, offers the possibility of generating predictive models capable of generalization to a large scale. In this case, the limiting factor of the study was based on the number of samples that have been diagnosed with IBD. Once these samples were identified, the negative class of our classifier was chosen randomly from all other samples in the data set. In order for the model to be robust, a balanced number of samples was chosen.

The dataset was divided into a train set and a test set. The train set was subsequently used for variable search and algorithm training. For the variable search, OTUs were agglomerated at the genus level. In addition, counts were log2 normalized before running the Feature Selection algorithms.

Four different strategies were used for feature selection, all of them independent of the Machine Learning models. The features selected by each of the FS strategies were the inputs for the two selected ML algorithms. The goal of this process was to select a subset of features, without altering the original representation of the data. Therefore, redundant and noisy variables were removed from the dataset. By selecting strategies independent of the classification algorithms, a comparison can be made in the performance of the algorithms, for further biological interpretation.

A nested resampling was used for the training of the models. The characteristic of this process is the presence of an independent internal cross-validation (2/3 for training and 1/3 for validation) for the selection of the best hyperparameters of each algorithm and an independent external cross-validation (5 repetitions of a 10-fold-CV) to evaluate the model in a general way. For each 10-fold-CV experiment, the samples were randomly divided into ten sets. Nine sets were used for training the model, and the remaining set was used for testing. The process was then repeated ten times such that each set was used once as a test set. The average performance of all 10 sets was reported as the final performance of the method. We repeated this process 5 times for each ML algorithm, and we presented the mean average of the 5 runs in the figures of the paper.

The performance of the different experiments was determined through the package "mlr" (Bischl et al., 2016). This package facilitates the design of machine-learning-based experiments, reducing the amount of scripting needed and providing a simpler and more manageable platform for development while facilitating reproducibility and replicability. Moreover, this package ensures that the execution of the machine learning algorithms follows the experimental design under the same conditions, thus allowing the comparison under equality of conditions. For the evaluation of the models, we used accuracy (to compare our findings with the state of the art) and the area under the receiver operating characteristic curve (AUC) to control for type I and II errors.

### 2.6. External Validation

The variability of metagenomic data, both biologically due to differences in population demographics and technicaly due to aspects such as sequencing platforms and sequencing depths, severely complicates the validation of predictive models in external databases. In this case, and in order to validate our models, as well as the variables found by the different FS strategies, two independent external datasets were downloaded from Morgan et al. (2012) and Gevers et al. (2014).

These datasets were chosen because they present two subtypes of IBD, Crohn's Disease (CD) and Ulcerative Colitis (UC). In this way, our models, without prior IBD subtype information, can be validated in two different subtypes.

As discussed above, due to the variability of the datasets, there are some genus in the training dataset that are not available in our validation cohorts. Therefore, in order to validate the information present in the variables identified by the FS algorithms, it was necessary to perform a retraining of the models from the variables available in those cohorts.

For model re-training, the features identified by each of the FS algorithms were selected. Subsequently, these features were intersected with the variables available in the validation cohorts. Only two subgroups of variables, those obtained by the FCBF techniques and those obtained by the kruskal wallis (K40) techniques, were taken to external validation. The main reason was the number of initial features (37 and 40, respectively). In this way, it is expected that the elimination of certain features will not notably influence the performance of the models.

Of the K40 subgroup, the cohort of Morgan et al. contained 21 characteristics, and Gevers et al. 22. As for the FCBF subgroup, Morgan et al. contained 9 and Gevers et al. 12.

## 3. Results

### 3.1. Presence of IBD Can Be Predicted by a Small Subgroup of Genus

**Figure 1:**
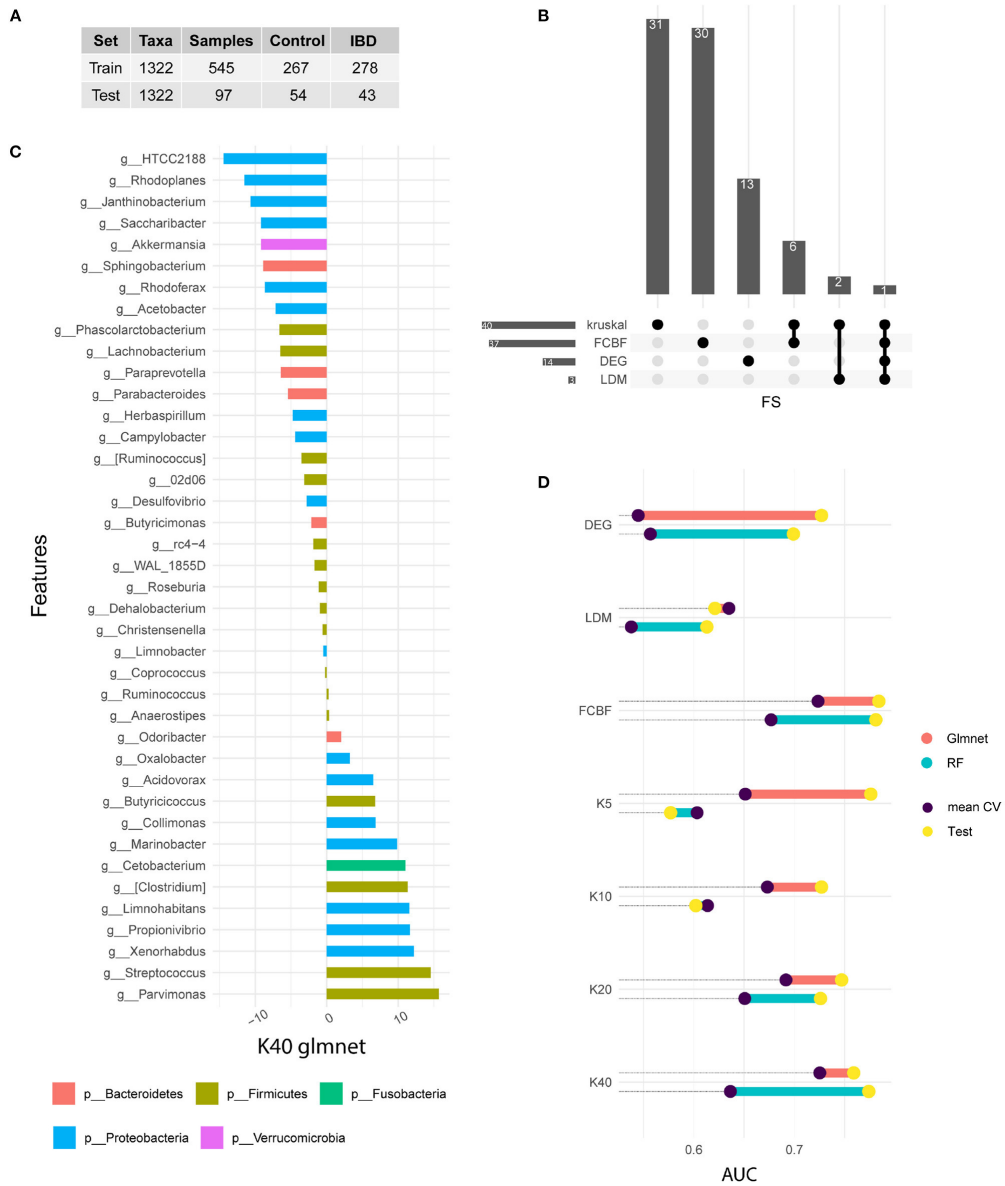
Signature identification. **(A)** Training and test data available from the American Gut Project for IBD samples. **(B)** Upset plot indicating the common features selected by each FS method. **(C)** Variable importance of the winning model measured after cross-validation. In this case, the 40 genera selected by the Kruskal Wallis method are shown. The bars represent the importance of the variables within the glmnet model. This was done by summing the betas of each variable over all iterations of the CV. **(D)** Comparison of the performance of the models in train and test. Note that the train value is the result of the arithmetic mean of the five iterations of the CV, while the test result is a single measure. The results of the glmnet and RF models are shown in AUC value.

We used four different strategies to identify distinctive features between the two different sample groups (IBD-positive and IBD-negative diagnosis). All feature selection methods used searched within the 1,322 different features, corresponding to genus level. The Figure 1A shows data ratios in both train and test sets.

We used Kruskal test as univariate method to rank genus according to their correlation with disease status. Different subgroups of genus were selected (5, 10, 20, and 40). The FCBF method was chosen as the multivariate filter method. This method identified 37 genus with low correlation between them and a high correlation with the target variable. On the other hand, 14 genus showed a differential expression between the two subgroups of samples. Finally, LDM-based feature selection identified three genus with significant differences. Figure 1B shows the features shared by each method. Kruskal (*n* = 40) and FCBF, which were the methods that identified the most features, have six genus shared only between them, while the vast majority were identified only by each method. On the other hand, differential abundance analysis identified 13 unique characteristics, while LDM shares two of its three characteristics with Kruskal. The genus Streptococcus is the only characteristic common to all four FS methods.

We used the output of each FS as input in ML-based classification models. Two types of supervised models were selected for classification analysis. Both models have been widely used in the field of omics analysis since are simple, fast and explainable. Figure 1D shows the performances achieved in both the train and test sets. The train results is the average of the performances of the 50 generated models (in purple), while test results (in yellow) shows the prediction of the 15% of samples excluded during data split.

Features identified by differential abundance and LDM do not show satisfactory performance during training, as it is shown in Figure 1D. On the other hand, both FCBF and Kruskal seem to identify features capable of adequately classifying patients according to their IBD diagnosis. Regarding kruskal, it seems that as features are added, glmnet model performs better. The same is not true for the RF algorithm, which experiences a drop on its performance after increasing from 20 to 40 features. We hypothesize that models with a higher number of genus such as FCBF and K40 perform better in both types of models and in both data subsets. Due to the heterogeneity and phenotypic complexity of IBD patients, it is necessary to include a large number of variables. This fact also leads to a higher risk of overfitting of the models, so validation with external cohorts is necessary.

In general, we observed test results achieving better performances than train results. This fact is easily explainable because the train performance is the mean of 50 results, while test performances corresponding only to a unique value. Therefore, lower values in some folds in CV experiments decrease the mean of the distribution. In that sense, it was considered appropriate to validate the models on a test subset.

We performed a normality analysis using the Shapiro-Wilk test with the null hypothesis that the data follow a normal distribution. The null hypothesis was rejected with values *W* = 0.9913 and *p* < 0.0003852 therefore it could be considered that our results did not follow a normal distribution. We performed a Bartlett test with the null hypothesis that our results were heteroscedastic. The null hypothesis was not rejected with a value for Barlett's K squared measure of 16.981 with 13 degrees of freedom and *p* < 0.2002. In this case, one of three conditions required for a parametric test does not hold and thus, consistent with both tests, we performed a non-parametric Friedman test with the Iman-Davenport extension assuming the null hypothesis that all models have the same performance.

The average rankings of the techniques compared are shown in the following table with Iman and Davenport statistic (distributed according to the F-distribution with 13 and 637 degrees of freedom: 43.26 and *p* < 9.88 10–79). Hence, glmnet model with 40 features selected by kruskal test is the control model.

After the test for choose the significantly better model, a Finner *post-hoc* procedure must be used in order to correct and adjust the *p*-values. Finner's procedure rejects hypothesis with a value ≤ 0.046, which means that the rest of the models but glmnet model trained with 37 features from FCBF are satistically significantly worse than the control model.

Figure 1C shows the variable importance of best model in the training set. This model corresponds to a glmnet model trained with 40 genus from kruskal-wallis test. Variable importance shows the sum of the betas over the 50 repetitions. The genera shown on the vertical axis correspond to the Greengenes taxonomic annotation performed by the American Gut Project. HTCC2188 and Parvimonas genus present the highest value of importance. Genera such as Rhodoplanes, Streptococcus, Xenorhabdus, Janthinobacterium, Propionivibrio or Limnohabitans also stand out. On the other hand, the genera Anaerostipes, Ruminocococcus, Coprococcus, Limnobacter, Christensenella, Dehalobacterium, and Roseburia are not important in the model.

Figure 1 also shows the phyla distribution through identified genus. We noted that Proteobacteria is the most representative phylum, with an abundance of 42.5%. In second place is the phylum Firmicutes with 40.5%, followed by Bacteroidetes with 12.5%, while Cetobacterium and Verrucomicrobia only present a single genus each.

Based on the sum of betas through the 50-fold CV experiment, we focused on the ranking of importance for each genus. We observe that Firmicutes and Proteobacteria occupy the top positions. It should also be noted that most of the genera belonging to the phylum Proteobacteria have a significant importance in the model, while eight of the 16 genera belonging to the phylum Firmicutes have an importance near zero. As for the phylum Fusobacteria and the phylum Verrucomicrobia, both are in positions of great significance.

The results observed throughout Figure 1 indicate that the metagenomic profile presents sufficient information for the classification of the samples according to their IBD diagnosis. It should be noted that no further information was included in the models, in the form of covariates, so the training of the models was performed only with information from 16S sequencing.

In addition, a correlation analysis was performed between the predictions of the selected models (in train and test) and certain cofounders that could affect the disease. Specifically, patient age, gender, alcohol consumption, BMI, antibiotic and probiotic intake and appendix removal were included in the analysis. The results of the correlation study in train and test are shown in Supplementary Figures S1, S2, respectively. In the train set, the variables corresponding to alcohol consuptiom (*p* = 0.038), antibiotic and probiotic intake (*p* = 1.6e-06) and appendix removal (*p* = 0.0014) presents significative values. In test subset, only alcohol consumption variable (*p* = 0.043) achieved significance.

Due to the variability and heterogeneity of the data generated by omics technology, it is necessary to validate the models in external cohorts. In the following section we show a validation in two external cohorts differentiating the samples according to its disease subtype.

### 3.2. Ulcerative Colitis and Crohn's Disease Shared Common Patterns at Genus Level

We carried out an external validation to interrogate if the identified genus has an informative value. In addition, we hypothesize that the identified subgroup is capable of identifying affected samples both in Ulcerative Colitis (UC) and Crohn's Disease (CD). In order to validate this hypothesis we selected two different cohorts to analyse the predictive value of our genus subgroup.

Although the type of IBD diagnosis was not specified in the AGP cohort, data from two external cohorts with two different subtypes of IBD were chosen. Gevers cohort (Gevers et al., 2014) includes samples of patients diagnosed with Crohn's Disease (CD), while Morgan cohort (Morgan et al., 2012) includes Ulcerative Colitis (UC) samples.

Based on the results in train and test sets, FCBF and K40 selected features were validated in external cohorts. Due to heterogeneity of sequencing platforms, not all genus are present in the external validation cohorts. In order to validate the general information in each genus subgroup, the intersect with available genus in both cohorts were made. Unfortunately, only twelve and nine genus of FCBF subgroup were present in Gevers and Morgan cohorts, respectively. Therefore, due to the significant loss of features, FCBF subgroup was not considered for external validation. In contrast, the subset found by K40 had 22 and 21 features in the Gevers and Morgan cohorts, respectively. Although the loss is also large in several metagenomic subsets, it was still considered appropriate to perform external validation bases on our hypothesis that shared features present sufficient information to obtain significant results in external cohorts.

**Figure 2:**
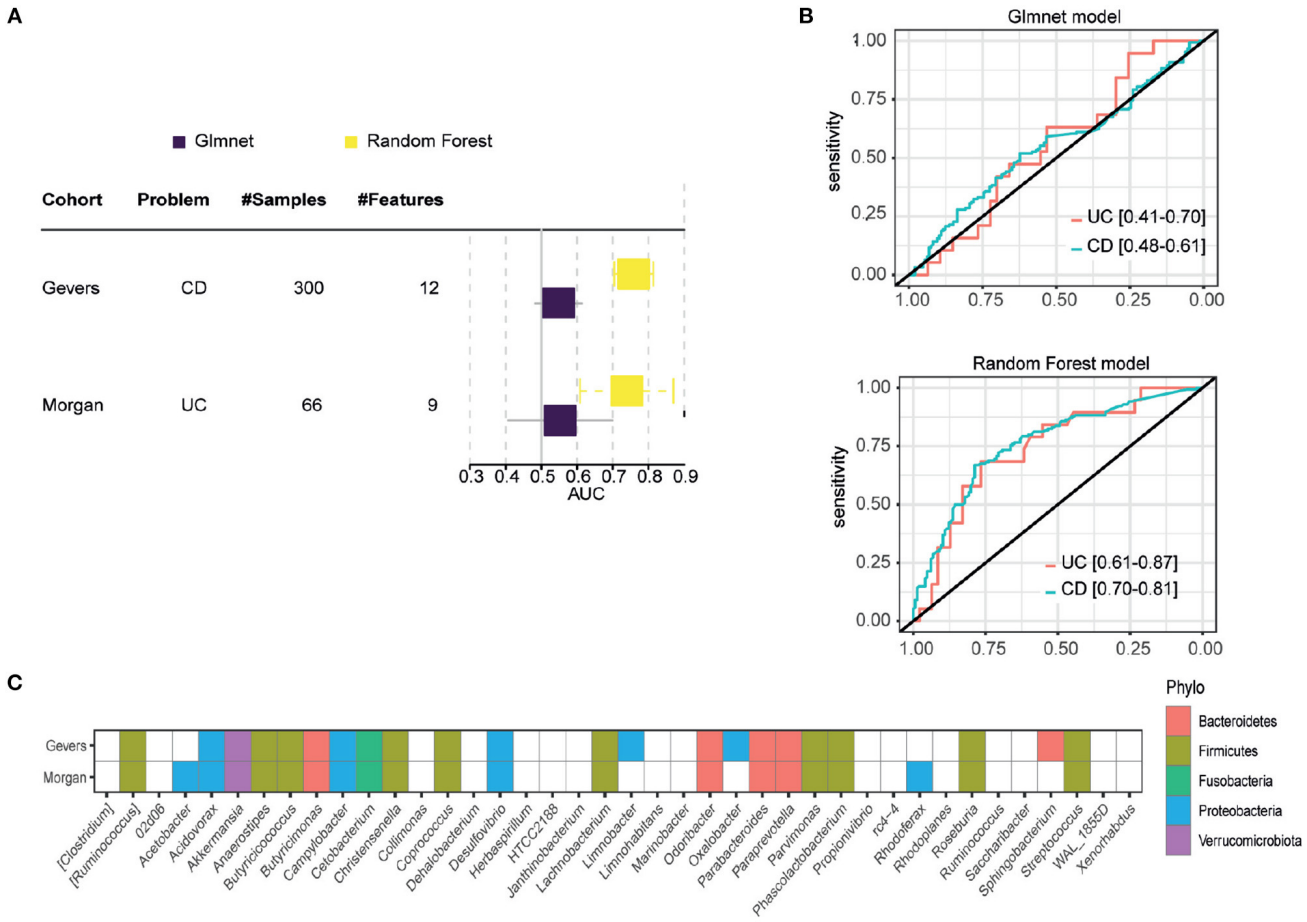
External validation results. **(A)** Results obtained in each of the external validation datasets, measured in AUC. **(B)** ROC curve of the RF model in the two external validation datasets. **(C)** Genes available in each external validation cohort, with which the models were re-trained.

Due the loss of genus, glmnet and RF models were re-trained in AGP cohort with the available genus. Figure 2A shows performances of both models in each cohort. Gevers and Morgan cohorts presents 300 and 66 samples respectively to validate the models. The used genus in external cohorts are shown in Figure 2C. On one hand, glmnet has low performance for both cohorts (0.5478 of AUC in Gevers; 0.5532 of AUC in Morgan) whereas RF model achieve better results in both (0.7588 of AUC in Gevers; 0.7391 of AUC in Morgan). ROC curves for RF models are shown in Figure 2B.

**Figure 3:**
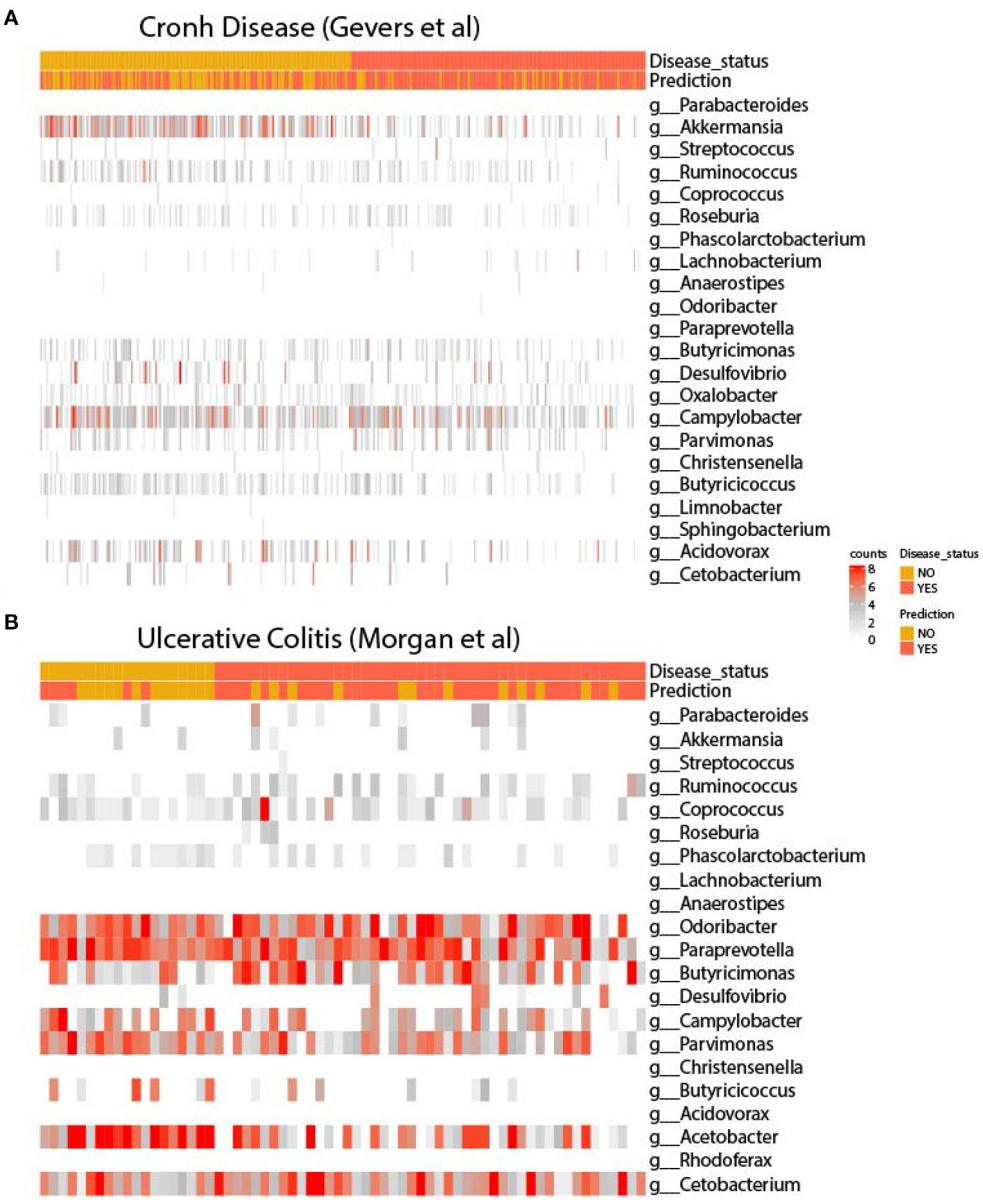
Heatmaps of the signature in external cohorts. Abundance of genera used for retraining in the cohort of **(A)** Gevers et al. and **(B)** Morgan et al. In both figures, genera are ordered according to model importance.

In order to infer microbiome distribution in the external cohorts, we plotted heatmaps in both cohorts (see Figure 3). Genus are sorted by model importance, while samples are sorted by disease status. Moreover, we included the prediction label for each sample from our model. Firstly, it can be seen that there is very little presence of some genera, mainly due to the heterogeneity of the sequencing platforms and their depth. Figure 3A shows how Parabacteroides, which is the most important genus in the model, is not present in the CD cohort. As for Akkermansia, there is a clear pattern of the presence of this genus in undiagnosed patients. Other two genera, such as Butyricicoccus and Acidovarax also exhibit a stratified distribution in the two subsets of patients. In terms of model predictions, a higher accuracy in predicting patients diagnosed with CD is observed. Despite the heterogeneity of the cohort, the model performance is considerably high.

As for the UC cohort, as shown in Figure 3B, the presence of the genera Parabacteroides, Coprococcus and Ruminocococcus seems to be more present in patients with the disease, while Parvimonas and Butyricicoccus seems to be more abundant in disease-free patients. As in the CD cohort, there is a very large heterogeneity between the training and validation cohort, with several important genera missing from the model. Even so, it appears that the model is able to accurately predict the presence of disease.

These results show the predictive capacity of our genus subgroup and the ML model to predict diverse subtypes of IBD. Although patients with IBD presents a wide range of symptoms, is clear that these subtypes share some metagenomic profiles.

## 4. Discussion

The results obtained in this work show the strong relationship between intestinal microbiome and IBD. Using FS and ML techniques, a relationship between different genus and the presence of the disease has been established. Furthermore, without the inclusion of any cofounder, high performance in predictions is observed, both in the set of train, test and external validation.

In the external validation, the glmnet model decreases its performance considerably, while RF obtains much better results. These results seem to indicate that the model obtained with glmnet in the training process presents a degree of overfitting. In addition, there could be non-linear relationships in the data, identified by the RF model and not by the glmnet model.

One aspect to consider is the use of genus as the taxonomic level to perform the search. Unlike the taxonomic level of species, which is more specific when it comes to establishing a diagnosis, the genus offers more robustness in the analyses. In addition, heterogeneity in sequencing platforms makes it difficult to standardize data across different cohorts. This is multiplied as we move down the taxonomic scale. Therefore, in our case, when training in a cohort such as the AGP cohort, where the sequencing depth is much greater than the validation cohorts, we consider that the use of genus as the taxonomic level is appropriate.

On the other hand, it has been observed that two subtypes of IBD such as UC and CD present common profiles in the microbiome. This is very interesting, because it makes it possible to search for common treatments in both subtypes. In CD, the pattern of genus Akkermansia suggest a clear protective action, which coincides with the results of the Magro et al. (2019). In general, there is no clear distribution of genus in the validation cohorts, which makes the use of ML techniques more valuable, as they are able to find complex non-linear patterns in order to obtain a high yield in previously unanalysed samples.

Motivated by these results, and in order to check which genus are related with each subtype, we performed an automatic analysis of the literature. We ran a script involving the use of Pubtator (Wei et al., 2013) annotations. This analysis allowed us to retrieve 162,674 documents in Pubmed associated with the organisms (including species and subspecies) discussed in this manuscript of which 140,646 (86%) also included at least one disease related MeSH term (a total of 8,164 different MeSH terms were identified). We subsequently focused on the identification of the co-citation of the bacteria of interest and the conditions of interest, Inflammatory Bowel disease (IBD), Crohn's Disease (CD) or Ulcerative Colitis (UC), using their MeSH associated terms (D015212, D003424, and D003093, respectively). We were able to identify a total of 21,544 (13%) documents co-citing these diseases and the identified microorganisms. IBD was the most co-cited term appearing in 10,611 papers (being the 26th most highly co-cited disease associated with this set of microbes), followed by CD and UC with 6,160 and 4,773 documents, respectively.

Finally, it is important to note a number of limitations of this study. Firstly, in metagenomics studies there is increased heterogeneity between cohorts. As mentioned above, due to the different sequencing platforms, there is a lot of difference in the available cohorts. Being able to validate the results is extremely difficult under these conditions. This fact adds value to the results obtained in this work. Even so, it is noted that a standardization of the cohorts would enable a better performance of the model. Moreover, the need to re-train the model due to the absence of different genders greatly limits the predictive capacity. On the other hand, the AGP cohort was used as a training set, which, although it has a large sample size, is not properly labeled, so it is possible that patients with very different degrees of the disease were labeled in the same way.

## 5. Conclusions

Here we have demonstrated how microbiome data can be used to predict IBD diagnosis through ML models. After microbiome signature search in IBD datasets, without subtype specifications, our model is able to predict IBD subtypes. These results says that the two subtypes of IBD such as Ulcerative Colitis and Cronh Disease have similar microbial patterns. Commons drugs and/or probiotics treatments can be works in both subtypes.

Ongoing efforts to investigate the roles of these microbes in IBD will be enable substantial improvements in early diagnosis and personalized treatments. Moreover, deeper examination of meta-cohort analyisis must be addressed in metagenomic field, in order to build most robust ML models.

## Data Availability Statement

Publicly available datasets were analyzed in this study. This data can be found here: http://ftp.microbio.me/AmericanGut/.

## Author Contributions

JL-B and GL-C: conceptualization. JL-B, CF-L, and JS: analysis conceptualization. JL-B: analysis pipeline, machine learning, and writing—original draft preparation. JL-B, CF-L, and GL-C: formal analysis. GL-C: text mining anlysis. CF-L, JS, and GL-C: review of drafts and supervision. All authors have read and approved the manuscript.

## Funding

CF-L's work was supported by the Collaborative Project in Genomic Data Integration (CICLOGEN) PI17/01826 funded by the Carlos III Health Institute from the Spanish National plan for Scientific and Technical Research and Innovation 2013-2016 and the European Regional Development Funds (FEDER)–A way to build Europe. JS's work was funded by the Ramón y Cajal grant (RYC2019-026576-I) funded by Ministry of Science and Innovation of the Spanish government. GL-C's work was supported by a grant from the Biotechnology and Biological Sciences Research Council (BBSRC grant BB/S006281/1) and open access publication fees were supported by Queen's University of Belfast UKRI block grant.

## Conflict of Interest

The authors declare that the research was conducted in the absence of any commercial or financial relationships that could be construed as a potential conflict of interest.

## Publisher's Note

All claims expressed in this article are solely those of the authors and do not necessarily represent those of their affiliated organizations, or those of the publisher, the editors and the reviewers. Any product that may be evaluated in this article, or claim that may be made by its manufacturer, is not guaranteed or endorsed by the publisher.
