# Supplementary material for: Machine Learning Based Microbiome Signature to Predict Inflammatory Bowel Disease Subtypes
Source: Front Microbiol. 2022 May 17;13:872671. doi: 10.3389/fmicb.2022.872671 (PMC9157387; doi:10.3389/fmicb.2022.872671)
Supplement: Supplementary file 1 [file Data_Sheet_1.PDF]

Supplementary Material

1 SUPPLEMENTARY DATA

1.1 Figures

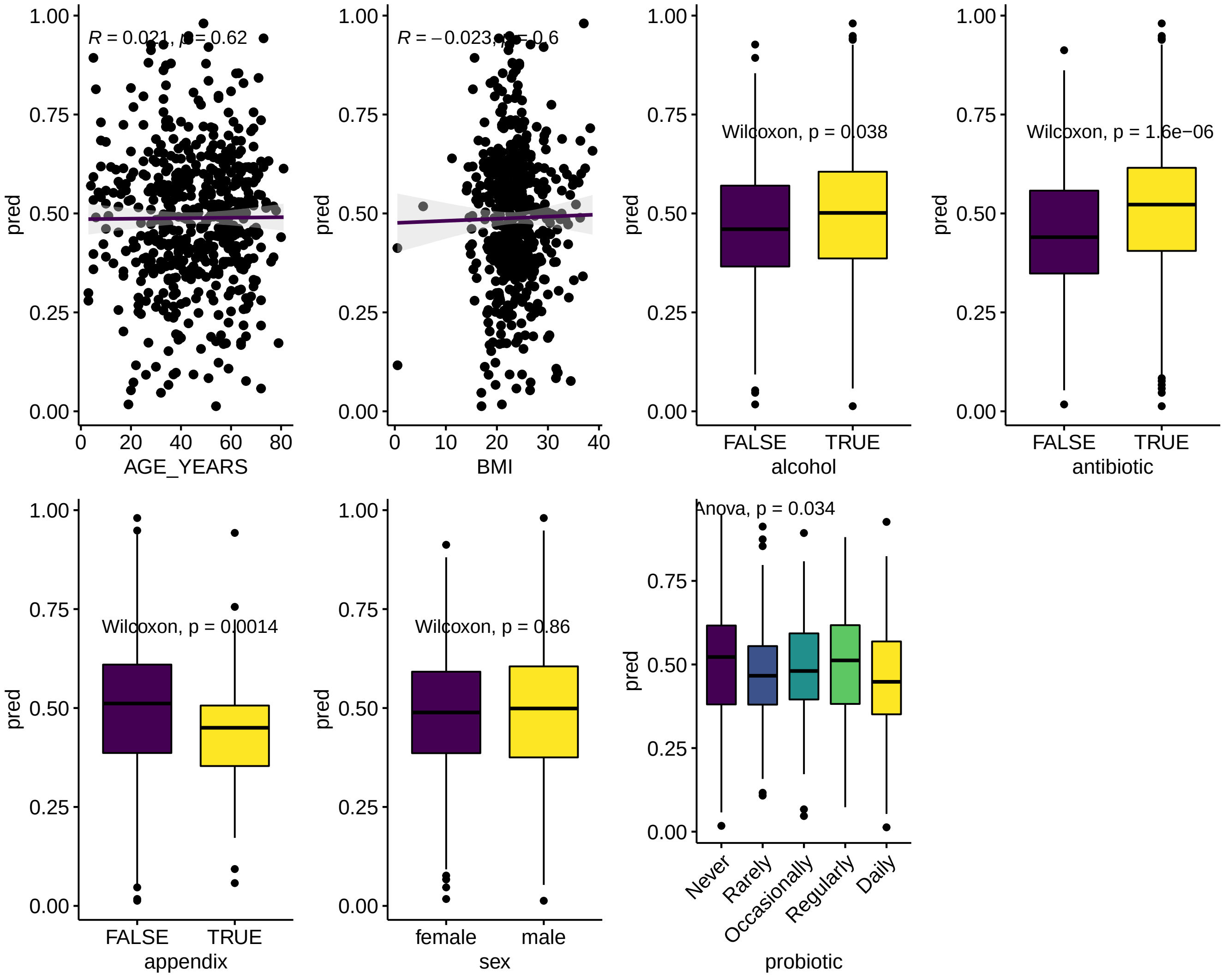

Figure S1. Cofounder analysis in train set.

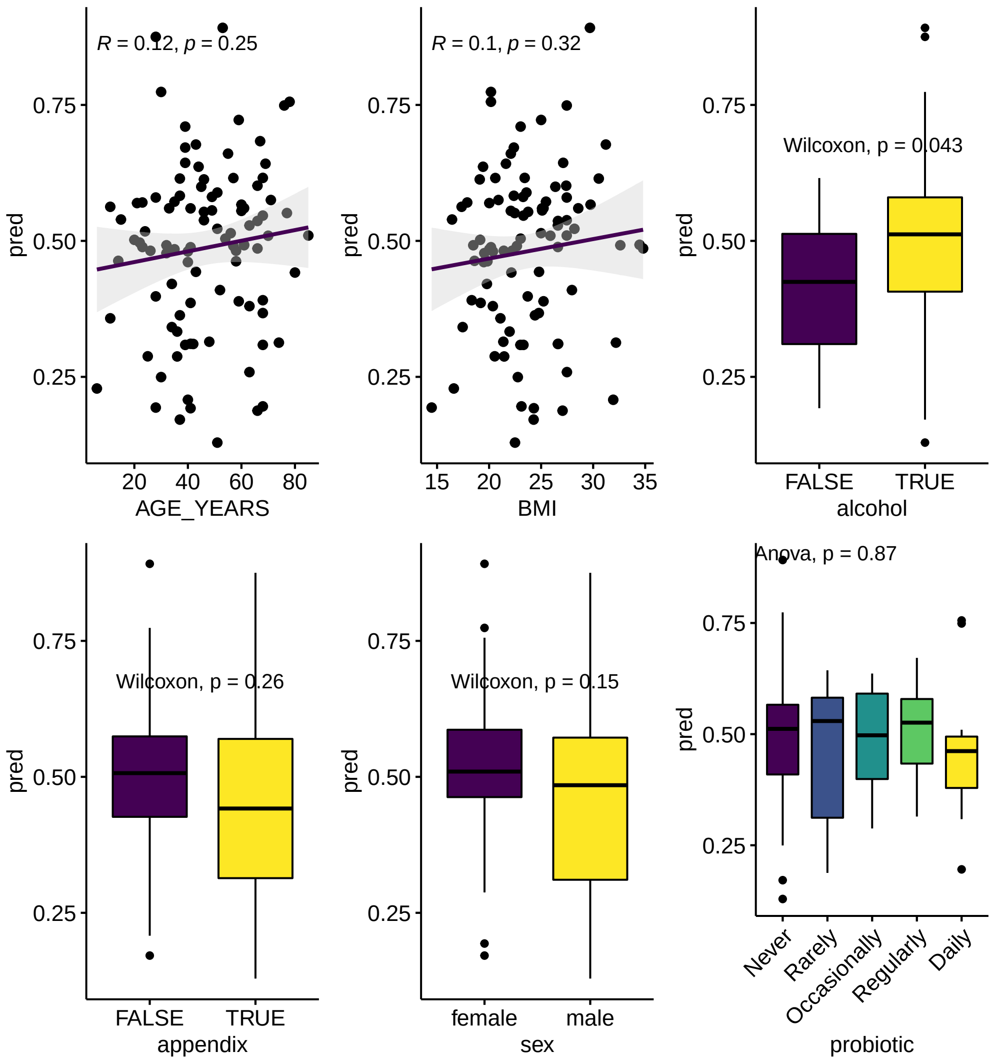

Figure S2. Cofounder analysis in test set.
